# Russian Petroleum

**Published:** 1884-07

**Authors:** 


### RUSSIAN PETROLEUM.

*ENGINEERING* gives some interesting facts in regard to the present conditi n of the manufacture of refined petrcleum from the oil obtained at Baku, in Southeastern Russia, near the Caspian Sea. Our readers know already that the Baku wells are ve:y productive, the yield exceeding that of the Pensylvania wells in their best days, and immense refineries have been built on the spot, for utilizing the oil as it flows from the wells. The largest of these establishments is that of the Nobel Brothers, which, although compactly planned, occupies a tract of ground a mile square, and sends out something like two thousond tons of marketable oil a day. A large portion of this oil is in the form of heavy residue, which is used for fuel by hundreds of of steamers on the Caspian Sea and the River Volga, and in most of the locomotives on the railways of Southeastern Russia. The heating effect of the petroleum fuel is said to be three times as great as that of an equal weight of coal, and it is made to burn without smoke in the improved furnaces, while the expenses of hauling and transportation are very much less than those incident to the use of coal. The kerosene, or ordinary illuminating oil, forms a much smaller proportion of the raw Russian petroleum than of the American; our refineries extracting 75 per cent. of kerosene from the same qnantity of crude oil which at Baku yields but 27 per cent. The quality of the Russian kerosene is said, however, to be somewhat superior to that exported from the United States, and the raw materials is so abundant and cheap that the Nobel standard kerosene is sold at the works for two cents a gallon. Kerosene being the most rapidly sold of the petroleum preducts, the smaller distillers, who have not enterprise enough to seek a market for the heavier and lighter portions of the oil throw them into the sea, reserving only enough of the heavy oil to burn under their dis illing retorts and in the furnaces of their steam-pumps; but the largest firms, with the economy characteristic of those who know both how to gain and to use wealth, utilize nearly everything, refining the naphtha, which they sell in Russia for use in various industrial processes, and extracting from the heavy portions a very good lubricating oil, which is sold all over Europe. To carry out still further this economy in manufacture, an attempt has been made to separate vaseline from the oil, and it is intended soon to establish a factory for making paraffine candles, and perhaps also for carrying on such delicate processes as that of extracting analine, and converting it into the costly dyes so much in use.

